# Supplementary material for: Primacy of vision shapes behavioral strategies and neural substrates of spatial navigation in marmoset hippocampus
Source: Nat Commun. 2024 May 14;15:4053. doi: 10.1038/s41467-024-48374-2 (PMC11093997; doi:10.1038/s41467-024-48374-2)
Supplement: Supplementary file 1 — Supplementary Information [file 41467_2024_48374_MOESM1_ESM.pdf]

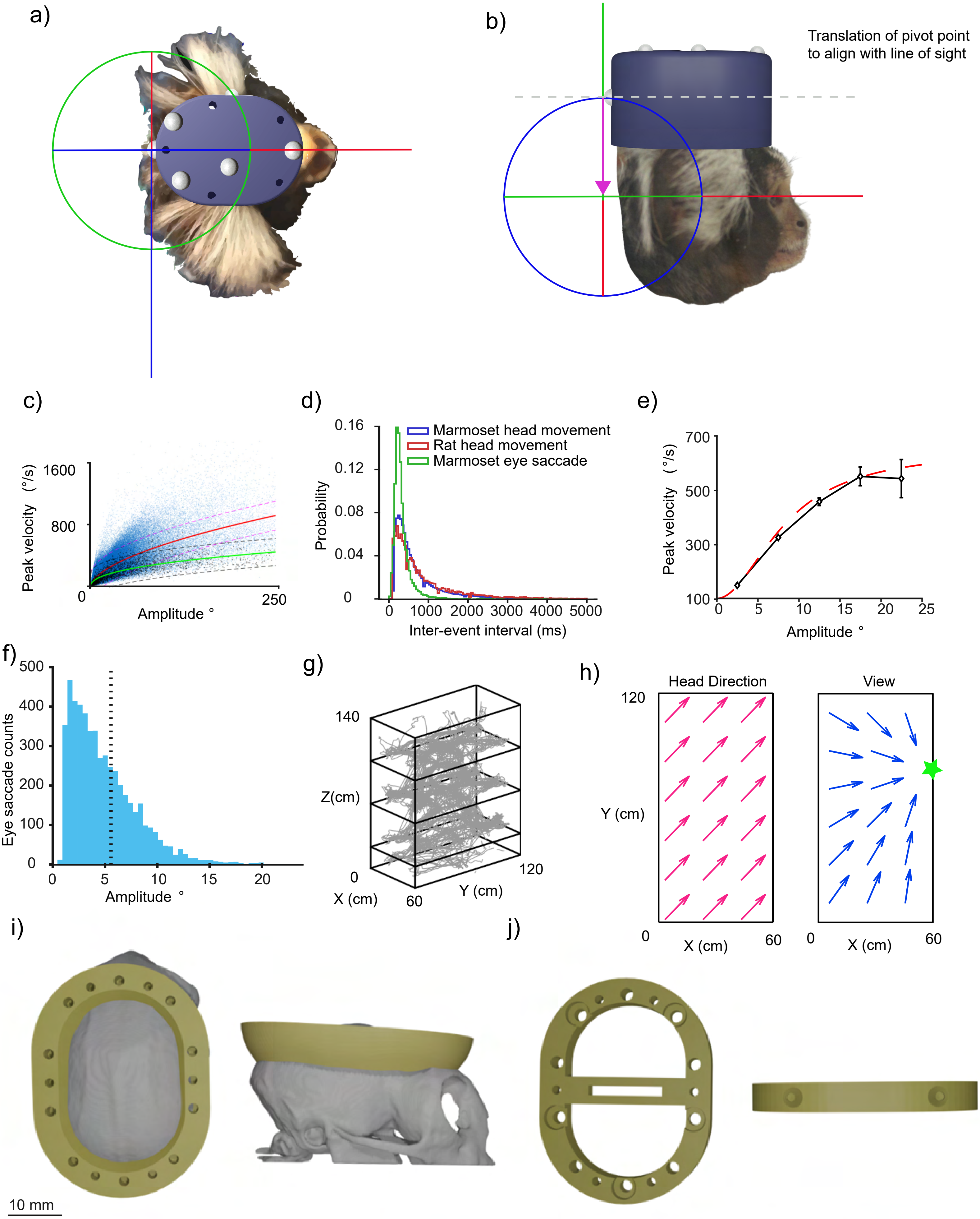

a)
b)
Translation of pivot point to align with line of sight
c)
Peak velocity (°/s)
1600
800
0
250
Amplitude °
d)
Marmoset head movement
Rat head movement
Marmoset eye saccade
Probability
0.16
0.12
0.08
0.04
0
0 1000 2000 3000 4000 5000
Inter-event interval (ms)
e)
Peak velocity (°/s)
700
500
300
100
0 5 10 15 20 25
Amplitude °
f)
Eye saccade counts
500
400
300
200
100
0
0 5 10 15 20
Amplitude °
g)
140
Z(cm)
0
X (cm)
60
Y (cm)
120
h)
Head Direction
View
120
Y (cm)
0
X (cm)
60
0
X (cm)
60
i)
j)
10 mm

Supplementary figure 1 (a) View from the top showing the alignment of the roll axis (red) with the visual axis, assuming a cyclopean eye. **(b)** Diagram illustrating the position of the pivot point (origin of rotation angles). The position of the pivot point (originally placed in the position of the most posterior retro-reflective marker) is linearly translated downwards, along the vertical axis to align with the subject's visual axis. **(c)** Main sequence (amplitude vs peak velocity) of head movements and Naka-Rushton function fit, for marmoset (blue markers, red line) and rat (black markers, green line); dashed lines correspond to 95% confidence intervals. **(d)** Distribution of inter-event interval (bin width = 50ms) for marmoset and rat head movements (blue and red distributions respectively) and marmoset eye saccades (green distribution). **(e)** Main sequence (amplitude vs peak velocity) of marmoset eye saccades, error bars convey 95% confidence intervals. The dashed line corresponds to a Naka-Rushton function fit. **(f)** Distribution of marmoset eye saccade amplitudes, dashed line placed at 5°. **(g)** Example trajectory traces (gray) of the 3D position during a recording session for subject C. **(h)** Illustration based on a previous publication[1], displays the vector direction differences between head direction and view encoding. **(i)** 3D model of the recording chamber ring placed on the skull of the marmoset. **(j)** 3D model of the spacer that houses the electrode connector (omnentics 36pin), the spacer is placed on top of the chamber ring and it serves as a head-fixation base.

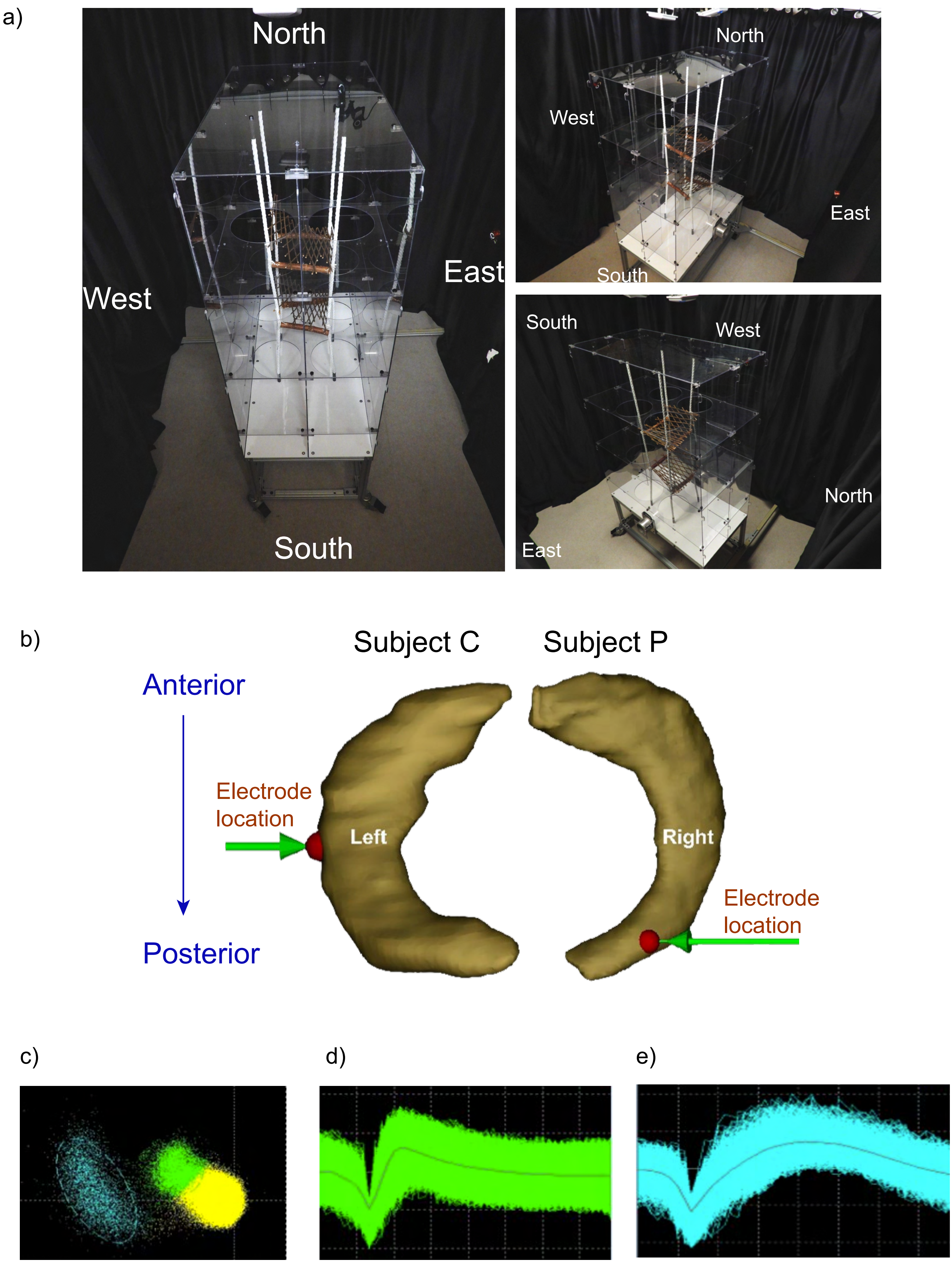
a)
North
West
East
South
North
West
East
South
South
West
North
East
b)
Subject C
Subject P
Anterior
Posterior
Electrode location
Left
Right
Electrode location
c)
d)
e)

Supplementary figure 2 (a) Pictures from multiple perspectives of the 3D maze with views of the laboratory space **(b)** MRI and micro-CT based 3D reconstruction of the electrode recording sites for subject C (left hippocampus) and subject P (right hippocampus). **(c)** Screenshot of a sorting session of one channel using Plexon software (Offline Sorter, Plexon Inc., Texas, USA). PCA view is displayed with labelled clusters, blue is a single unit, green is multi-unit activity, and yellow is the noise unit. **(d)** Multi-unit activity spike waveforms, aligned to the negative peak. **(e)** Single unit activity spike waveforms, aligned to the negative peak.

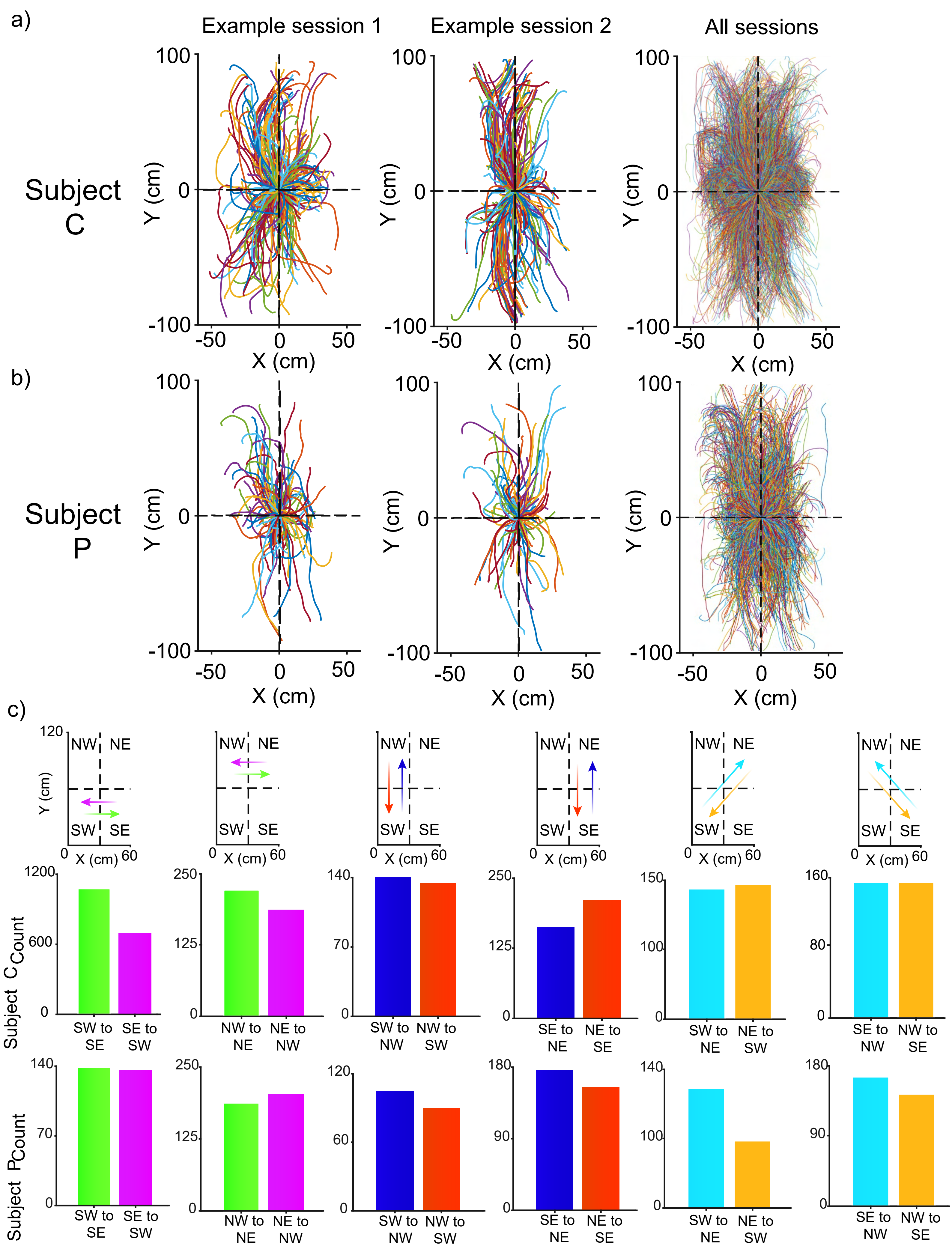
a)
Example session 1
Example session 2
All sessions
Subject C
b)
Subject P
Y (cm)
X (cm)
100
0
-100
-50
50
c)
NW
NE
SW
SE
120
60
Y (cm)
X (cm)
Subject C Count
Subject P Count
SW to SE
SE to SW
NW to NE
NE to NW
SW to NW
NW to SW
SE to NE
NE to SE
SW to NE
NE to SW
SE to NW
NW to SE
1200
600
250
125
140
70
150
100
160
80
90
180
60

Supplementary figure 3 (**a,b**) Individual trajectories travelled from example individual sessions (left and center panels) and all sessions (right panel). Each trajectory originates in the center (x=0,y=0) and the heading direction can be traced from that origin (**c**) Counts of complementary trajectories travelled along all possible paths between quadrants (SW= southwest, SE= southeast, NW= northwest, NE= northeast) across all trajectories across all sessions (Subject C = 26 sessions, Subject P= 33 sessions).

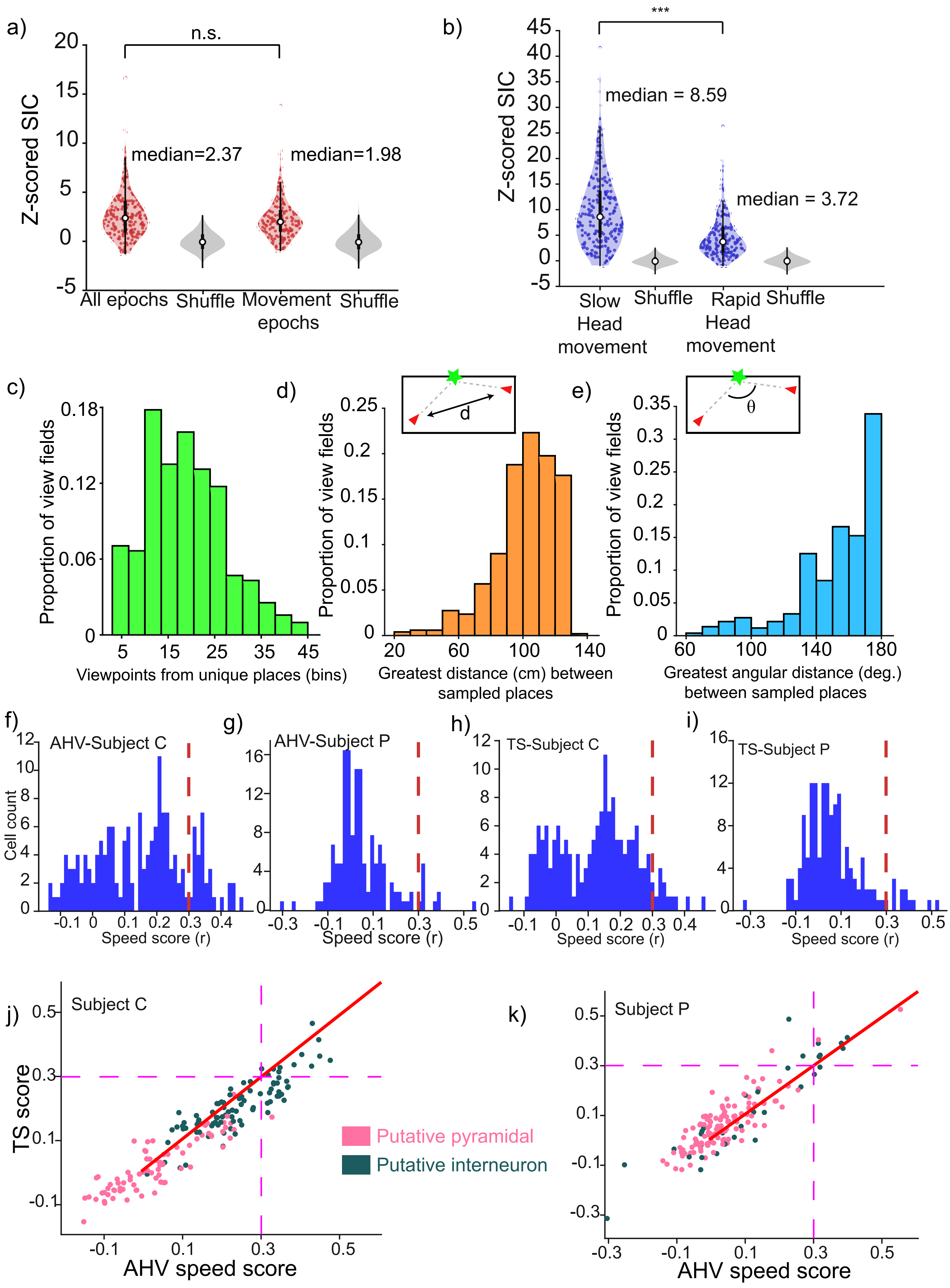

a)
n.s.
Z-scored SIC
median=2.37
median=1.98
All epochs
Shuffle
Movement epochs
Shuffle
b)
***
Z-scored SIC
median = 8.59
median = 3.72
Slow Head movement
Shuffle
Rapid Head movement
Shuffle
c)
Proportion of view fields
Viewpoints from unique places (bins)
d)
d
Proportion of view fields
Greatest distance (cm) between sampled places
e)
θ
Proportion of view fields
Greatest angular distance (deg.) between sampled places
f)
AHV-Subject C
Cell count
Speed score (r)
g)
AHV-Subject P
Speed score (r)
h)
TS-Subject C
Speed score (r)
i)
TS-Subject P
Speed score (r)
j)
Subject C
TS score
AHV speed score
Putative pyramidal
Putative interneuron
k)
Subject P
AHV speed score

Supplementary figure 4 (a) Distribution of spatial information content (SIC) during all epochs, movement epochs and shuffled controls. **(b)** Distribution of spatial information content (SIC) during epochs of no head movement, epochs of rapid head movement and shuffled controls. **(c)** Distribution of viewpoints from unique places per every view field. (**d)** Greatest Euclidean 3D distance between the unique sampled places per view field. (**e)** Greatest angular distance between view projections sampled from unique places per view field.**(f,g)** AHV speed scores for subject C and subject P respectively (bin width = 0.017), the dashed line indicates a speed score of 0.3. **(h,i)** TS speed scores for subject C and subject P respectively, the dashed line indicates a speed score of 0.3. **(j,k)** Speed score distribution for both AHV and TS labelled according to cell type for subject C and subject P respectively, (green = putative interneuron, pink = putative pyramidal), the dashed line indicates a speed score of 0.3.

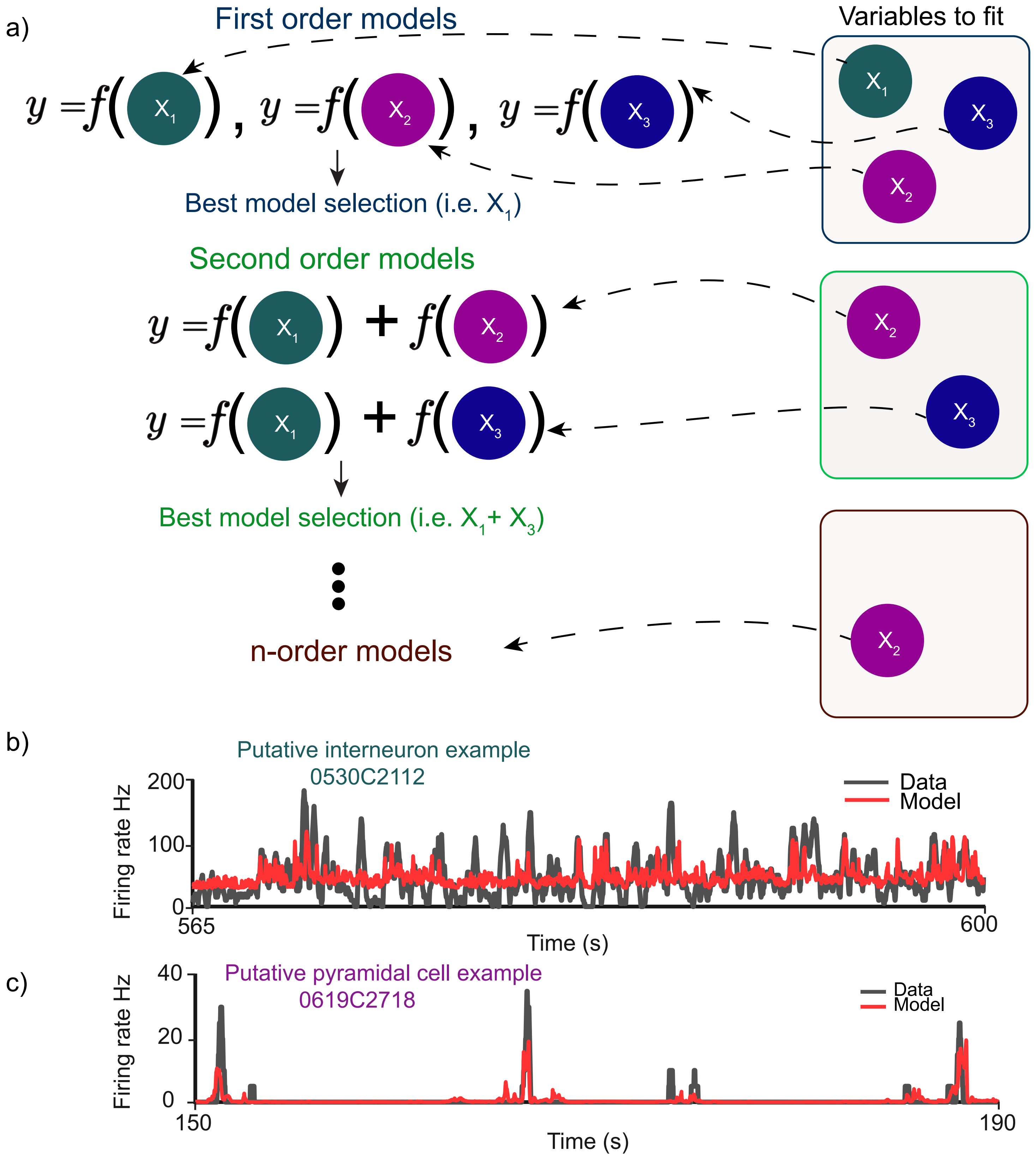

Supplementary figure 5 (a) Schematic describing the stepwise forward search GAM model selection process. **(b)** Real vs. predicted firing rate for a putative interneuron example cell (example cell from **Fig.6c**). **(c)** Real vs. predicted firing rate for a putative pyramidal example cell (example cell from **Fig.6a**).

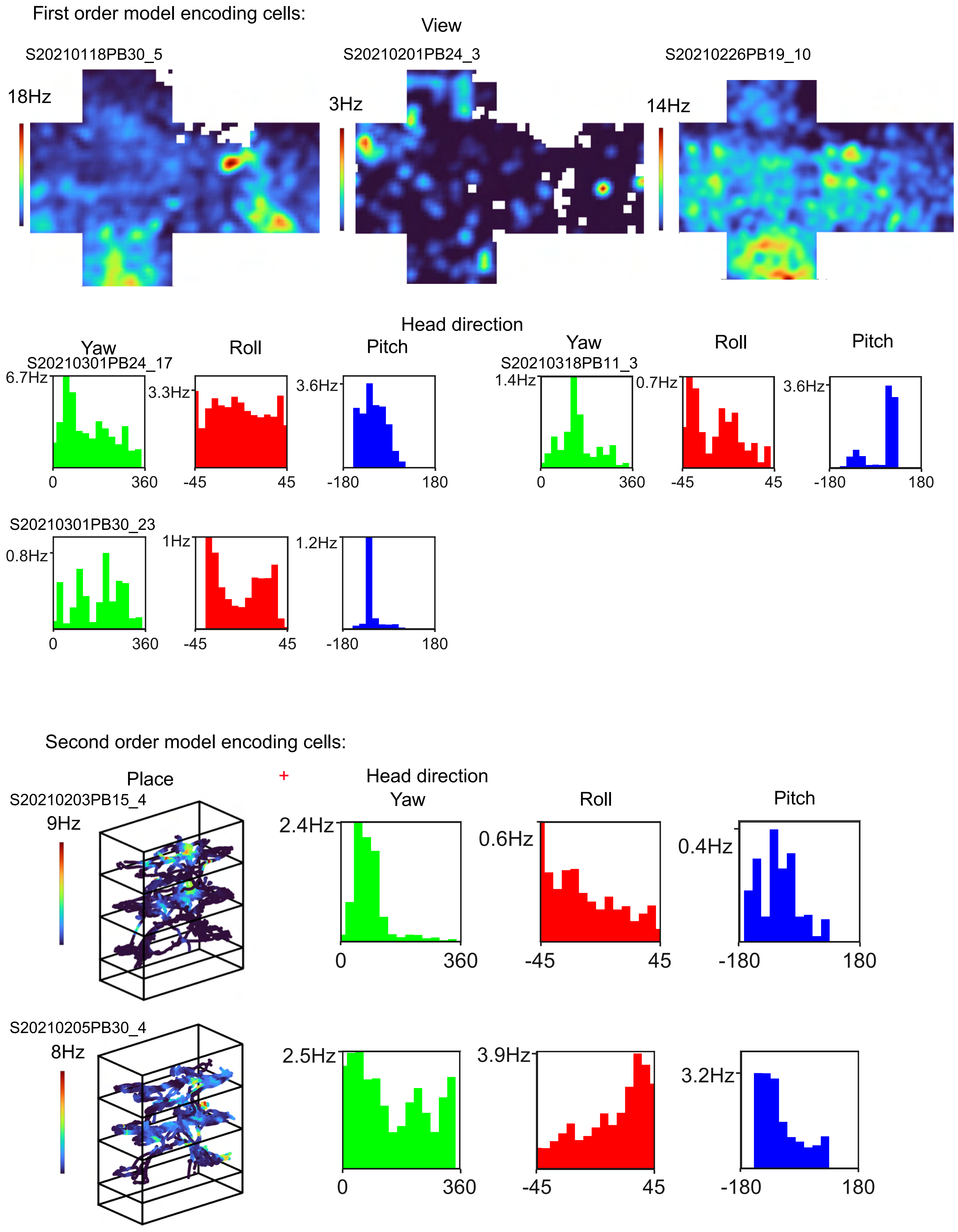
First order model encoding cells:
View
S20210118PB30_5
18Hz
S20210201PB24_3
3Hz
S20210226PB19_10
14Hz
Head direction
Yaw
Roll
Pitch
S20210301PB24_17
6.7Hz
3.3Hz
3.6Hz
0
360
-45
45
-180
180
S20210318PB11_3
1.4Hz
0.7Hz
3.6Hz
S20210301PB30_23
0.8Hz
1Hz
1.2Hz
Second order model encoding cells:
Place
+
Head direction
S20210203PB15_4
9Hz
2.4Hz
0.6Hz
0.4Hz
S20210205PB30_4
8Hz
2.5Hz
3.9Hz
3.2Hz

Second order model encoding cells:

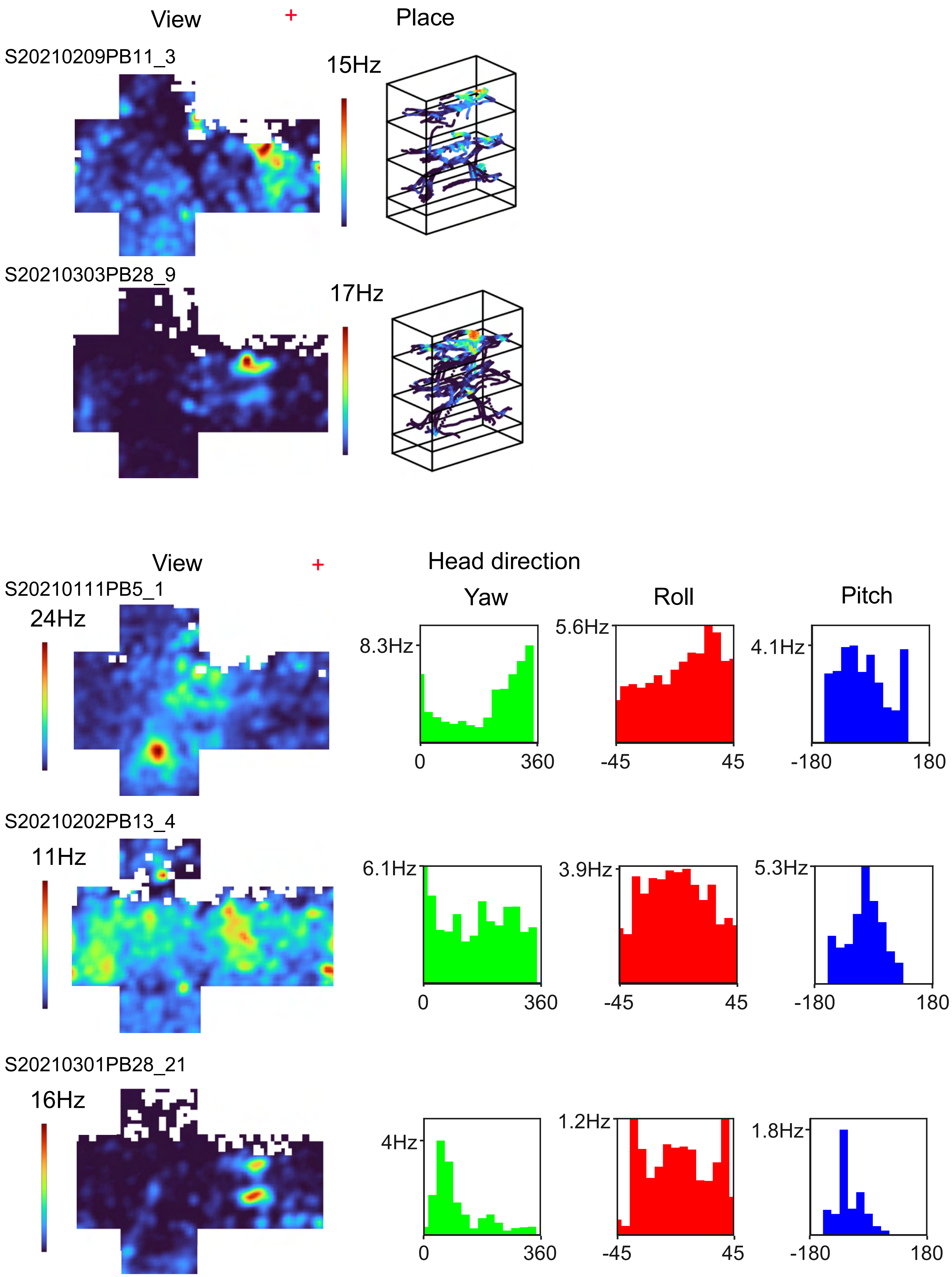

Third order model encoding cells:

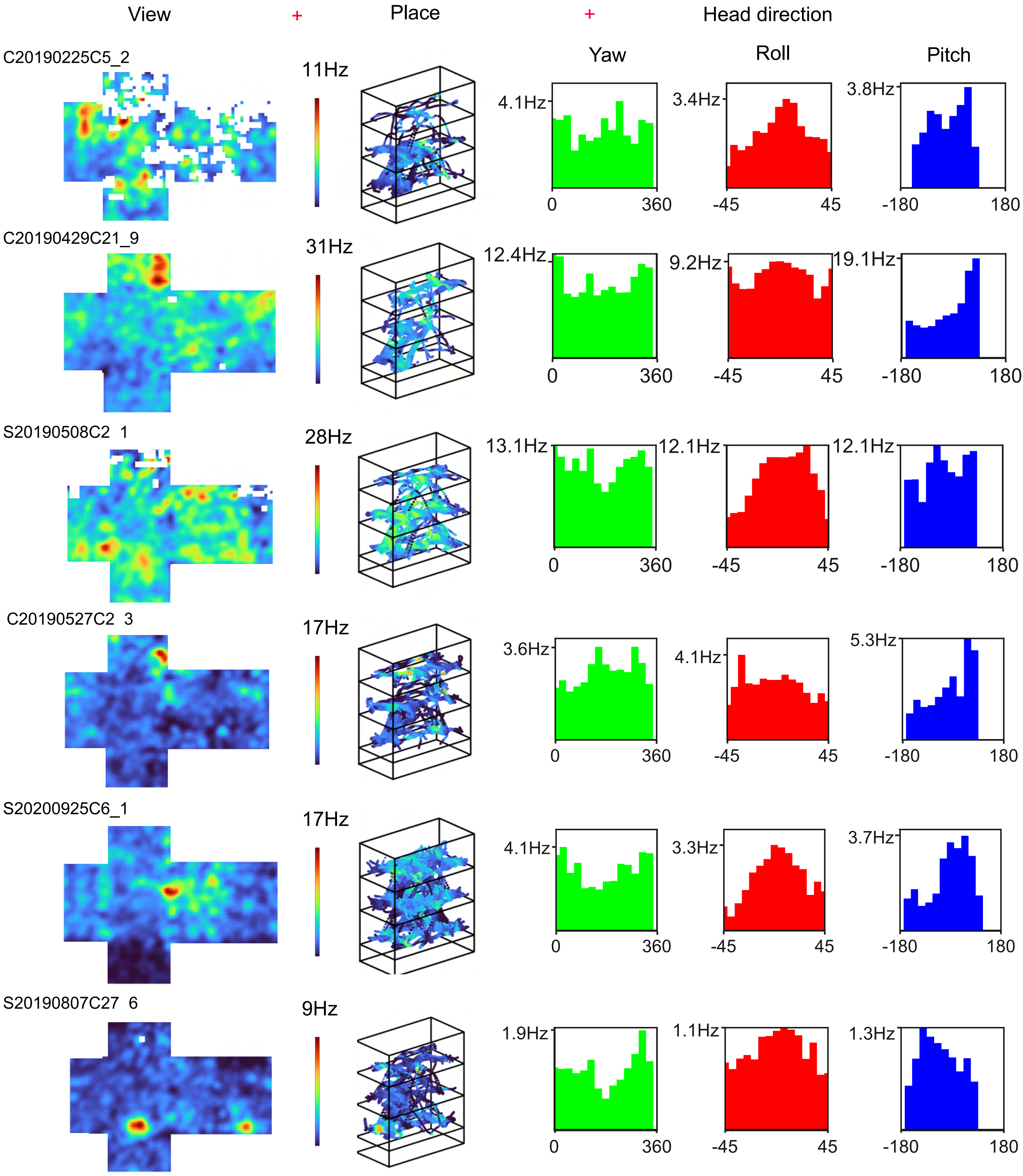

Third order model encoding cells:

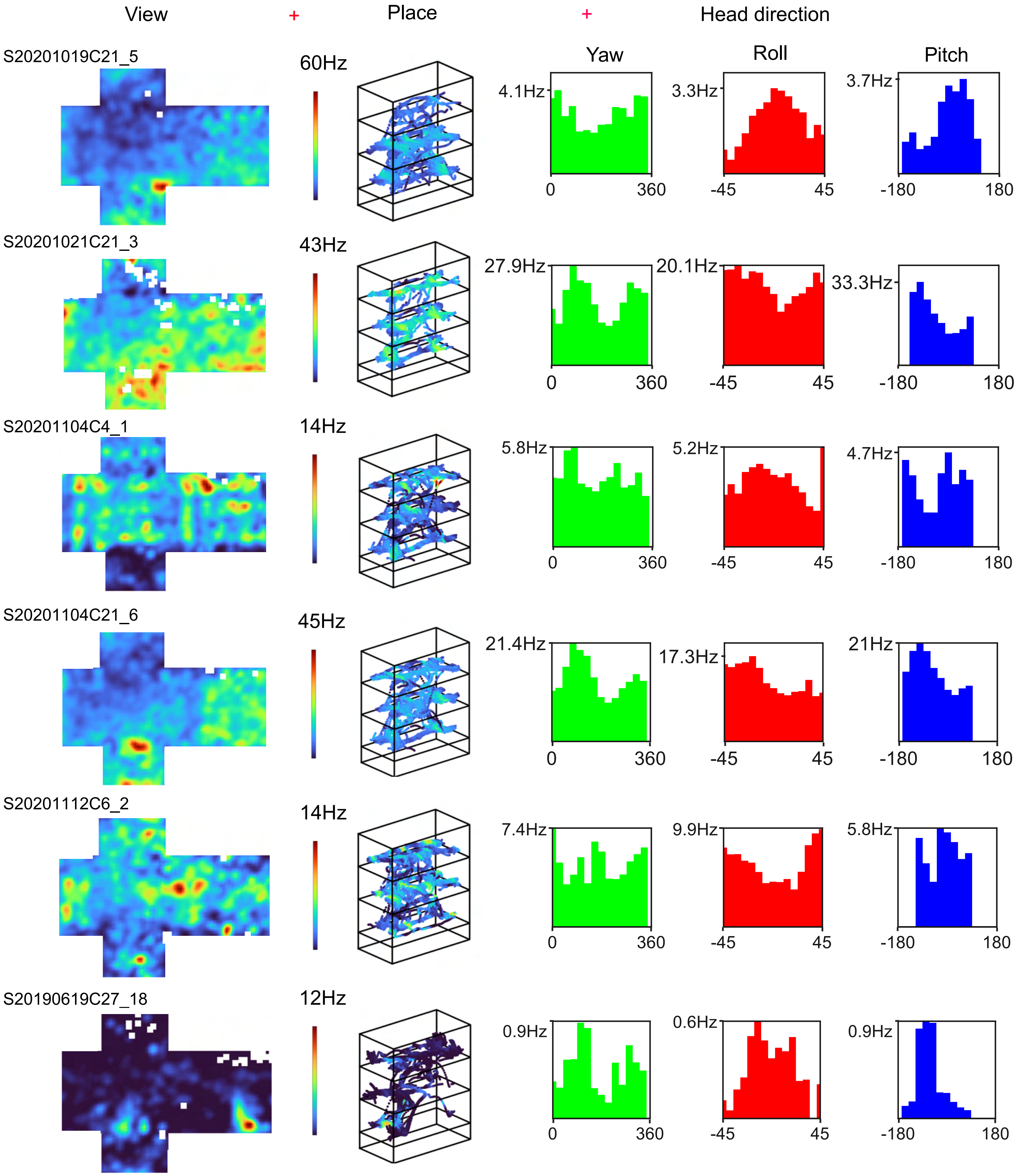

Third order model encoding cells:

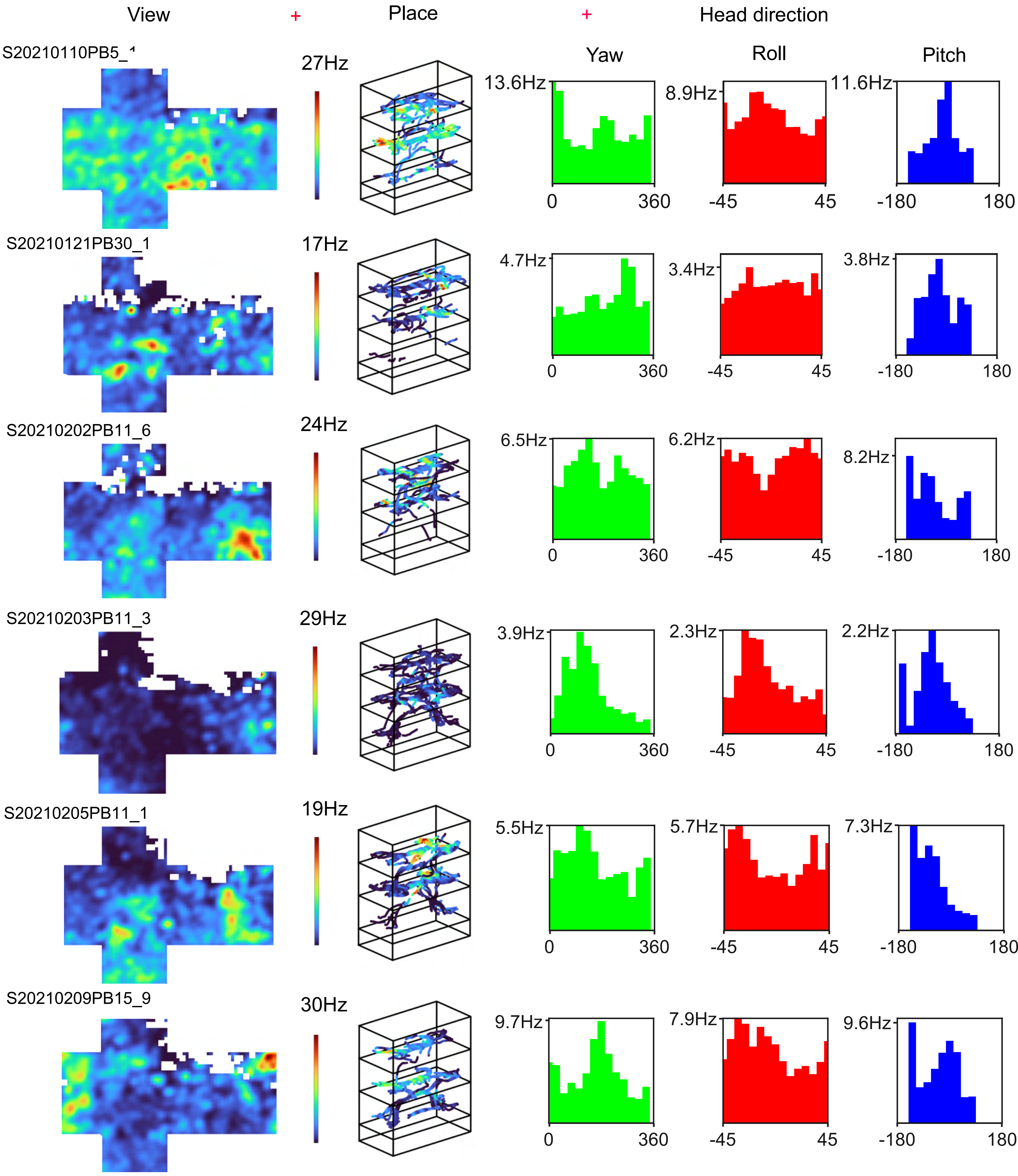

Third order model encoding cells:

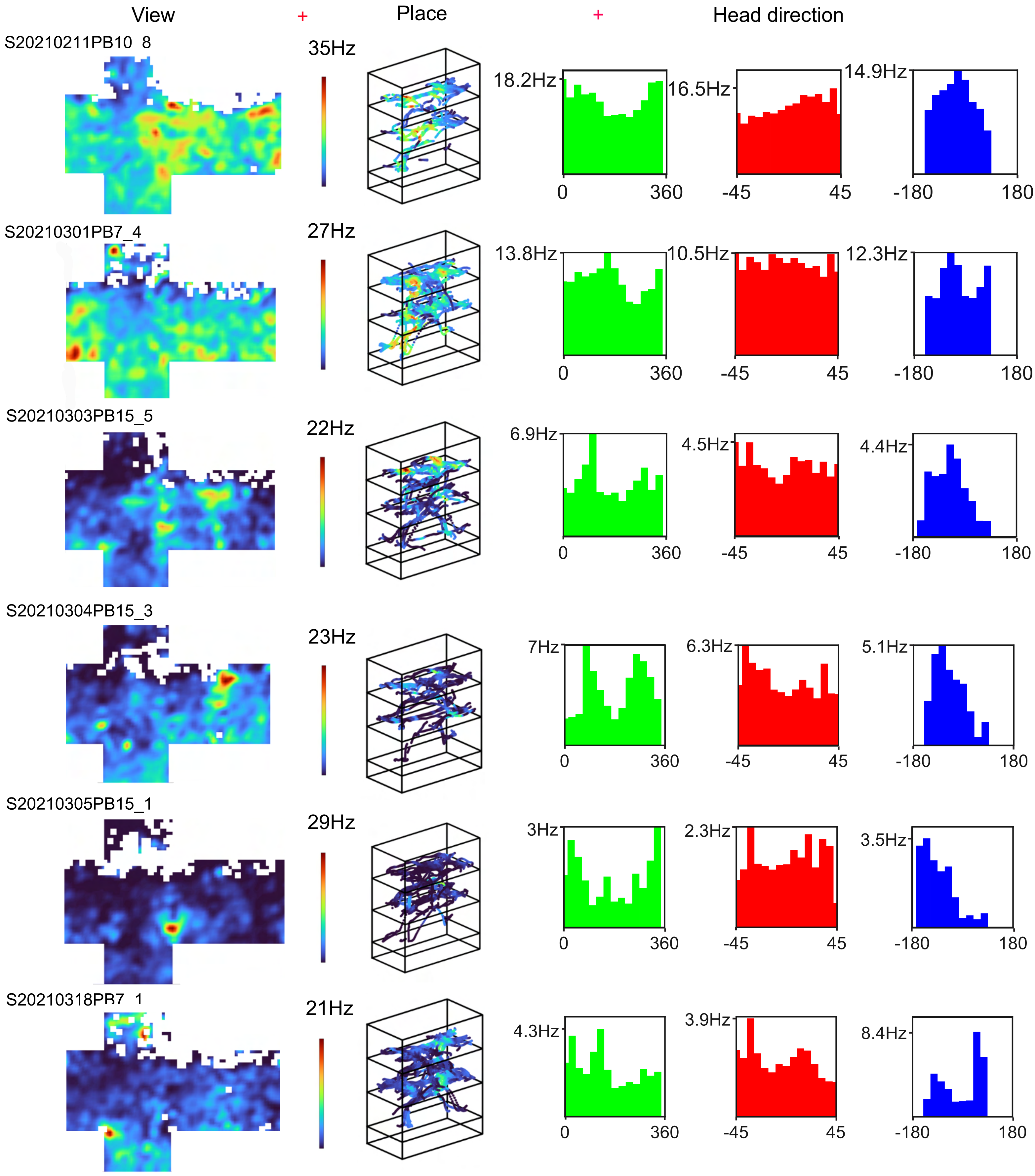

Supplementary figure 6 Additional single-cell examples are provided to illustrate the diverse types of encoding observed in putative pyramidal single neurons using the GAM model. Rate maps depicting view, place, and head direction are plotted to represent the corresponding behaviors encoded by individual neurons. In the case of cells encoding only one variable (first order models), a single rate map is observed. Conversely, in cells encoding multiple variables (second and third order models) multiple rate maps are displayed, indicating the specific behaviors being encoded. In head direction rate maps, firing rates corresponding to yaw angles (horizontal head direction) are color-coded as green (bin width = 24°), roll angles (lateral tilt) as red (bin width = 6°), and pitch angles (vertical axis) as blue (bin width = 24°). In the case of third order models, the view and place rate maps share the same color map, with the maximum firing rate indicated at the top of the color bar.

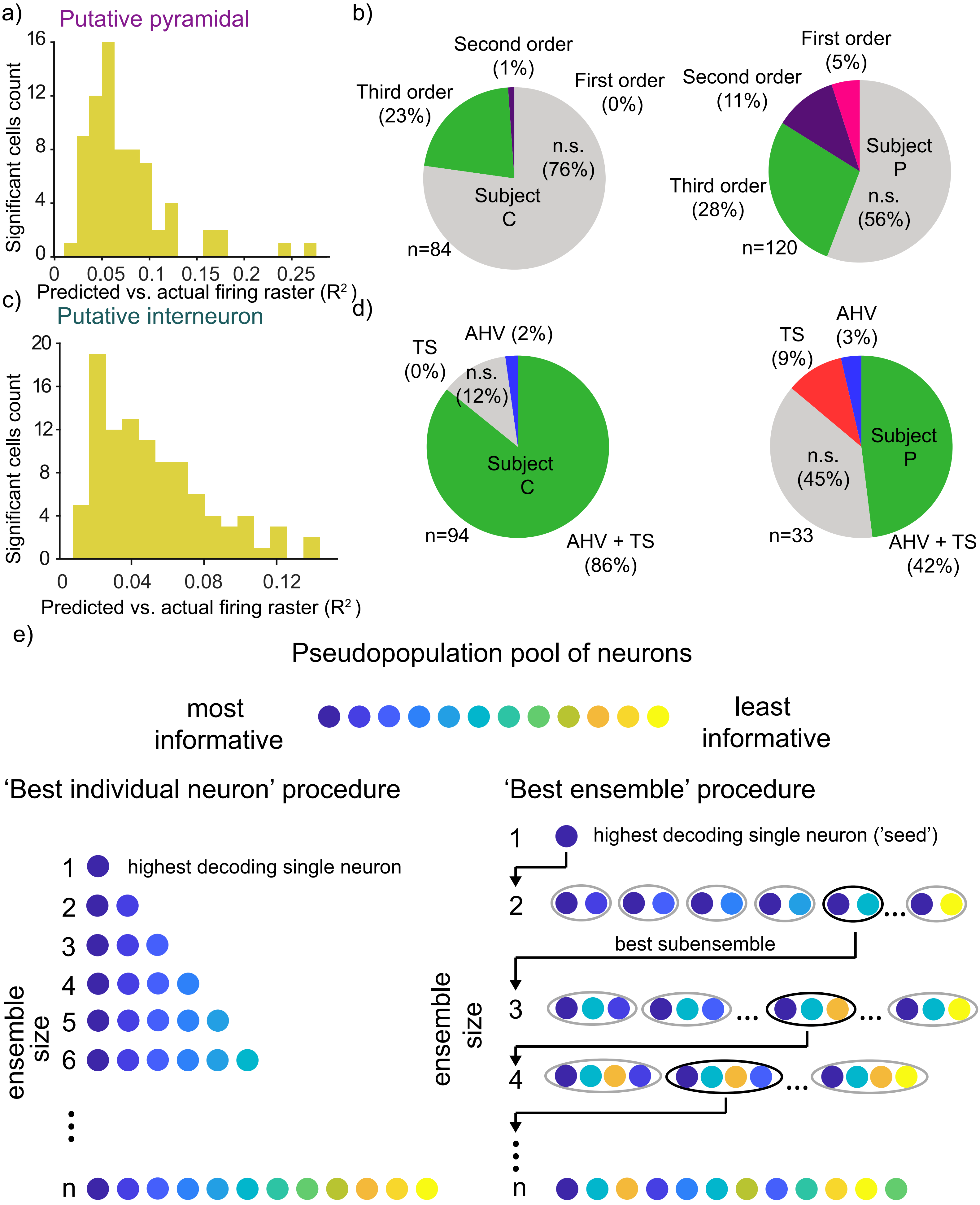

a)
Putative pyramidal
Significant cells count
Predicted vs. actual firing raster (R^2)
b)
Second order (1%)
First order (0%)
Third order (23%)
n.s. (76%)
Subject C
n=84
First order (5%)
Second order (11%)
Subject P
Third order (28%)
n.s. (56%)
n=120
c)
Putative interneuron
Significant cells count
Predicted vs. actual firing raster (R^2)
d)
TS (0%)
AHV (2%)
n.s. (12%)
Subject C
n=94
AHV + TS (86%)
TS (9%)
AHV (3%)
Subject P
n.s. (45%)
n=33
AHV + TS (42%)
e)
Pseudopopulation pool of neurons
most informative
least informative
'Best individual neuron' procedure
highest decoding single neuron
ensemble size
'Best ensemble' procedure
highest decoding single neuron ('seed')
best subensemble
ensemble size

Supplementary figure 7 (a) Average R^2 between predicted and real spike rasters of all significantly encoding putative pyramidal cells (bin width = 0.013 R^2). **(b)** Proportion of putative pyramidal encoding cells for subject C (left) and subject P (right). **(c)** Average R^2 between predicted and real spike rasters of all significantly encoding putative interneuron cells (bin width = 0.01 R^2). **(d)** Proportion of putative interneuron encoding cells for subject C (left) and subject P (right). Since each recording session is divided into 5 equally-timed folds, each predicted raster is a continuous time series approximately 8-12 min. long. **(e)** Schematic describing the best ensemble building procedure. (left) The most informative neuron is selected, as assessed by the single-neuron with the highest SVM decoding accuracy of place. (right) The most informative neuron is used as a seed to iteratively add units (looped through the remaining units) to the subsequent subensembles until the 'best ensemble' n-combination of neurons is obtained.

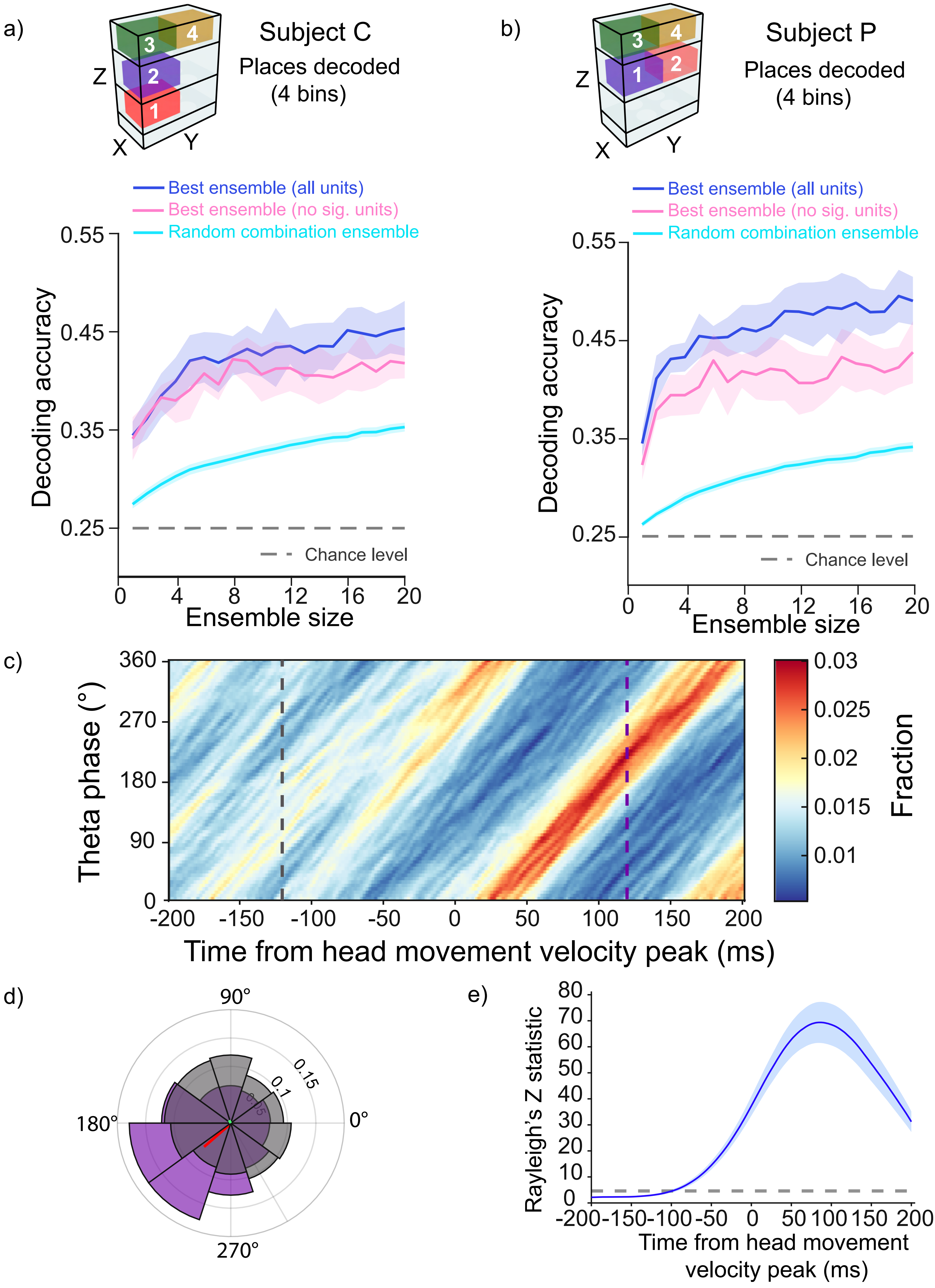

a)
Subject C
Places decoded
(4 bins)
Z
X
Y
3
4
2
1
Best ensemble (all units)
Best ensemble (no sig. units)
Random combination ensemble
Decoding accuracy
0.55
0.45
0.35
0.25
Chance level
0
4
8
12
16
20
Ensemble size
b)
Subject P
Places decoded
(4 bins)
Z
X
Y
3
4
1
2
Best ensemble (all units)
Best ensemble (no sig. units)
Random combination ensemble
Decoding accuracy
0.55
0.45
0.35
0.25
Chance level
0
4
8
12
16
20
Ensemble size
c)
Theta phase (°)
360
270
180
90
0
-200
-150
-100
-50
0
50
100
150
200
Time from head movement velocity peak (ms)
0.03
0.025
0.02
0.015
0.01
Fraction
d)
90°
180°
0°
270°
0.15
0.1
0.05
e)
Rayleigh's Z statistic
80
70
60
50
40
30
20
10
0
-200
-150
-100
-50
0
50
100
150
200
Time from head movement
velocity peak (ms)

Supplementary figure 8 (a,b) (top) 3D diagram of the binned place locations used to decode the subject's position, (bottom) blue and pink lines correspond to unfitted, mean decoding accuracy (y axis) as a function ensemble size (number of neurons, x axis), shaded area corresponds to 95% confidence intervals. Blue solid lines correspond to the best ensemble constructed from a pool of all recorded putative pyramidal neurons. Pink solid lines correspond to the best ensemble constructed from a pool of non significantly selective cells (as per GAM encoding analysis). R^2 goodness of fit value is reported. The cyan lines correspond to the mean decoding accuracy of a randomized combination of neurons (100 iterations), shaded area corresponds to 95% confidence intervals. The gray dashed lines correspond to chance decoding accuracy (1/4, 0.25). **(c)** Heatmap of mean phase concentration around a 400ms window centered on head movement peak velocity for an example channel during a recording session (~1hr) recording for n=2044 head movements, dotted gray line indicates -120ms, dotted purple line indicates +120ms **(d)** Phase distribution during -120ms (gray) and +120ms (purple) time epochs. Green and red lines indicate circular mean and vector length of the gray and purple distribution respectively. **(e)** Mean Rayleigh's Z-statistic for non-uniformity of circular data corresponding to n=59 recording sessions for a 400ms window centered on head movement peak velocity, shaded area represents 95% confidence interval. Gray dotted line indicates critical value for significance at $\alpha=0.01$.

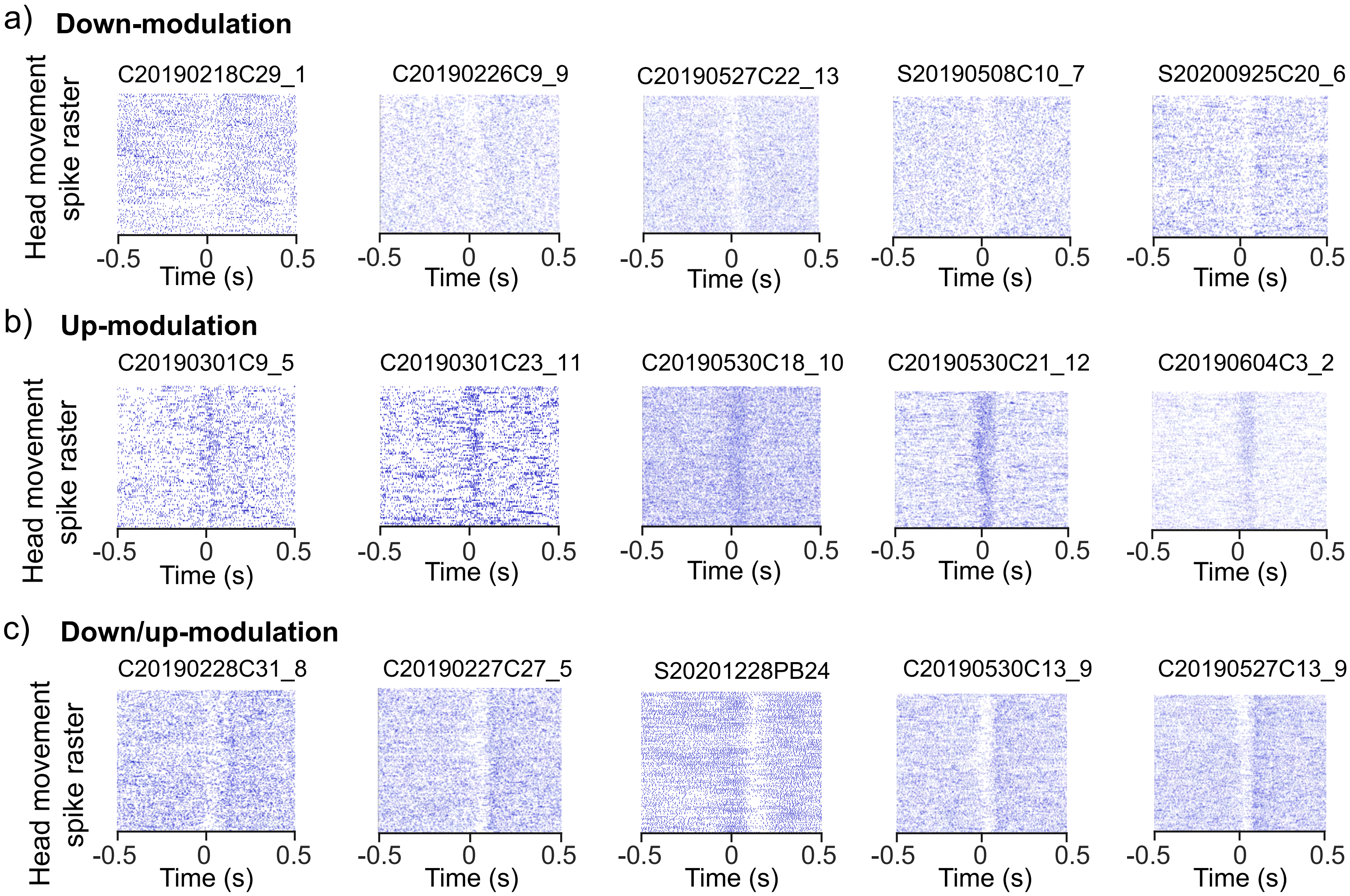

Supplementary figure 9 (a-c) Additional single-cell examples of down-modulated, up-modulated and down/up modulated cells respectively. Each raster plot corresponds to a single neuron and is aligned (time 0) with the peak velocity time of a head movement, where each row corresponds to one head movement event and each blue dot represents one neuronal spike, negative times indicate the neuron's firing before the peak velocity of a head movement, while positive times indicate the neuron's firing afterwards.

References

1. Franzius, M., Sprekeler, H. & Wiskott, L. Slowness and Sparseness Lead to Place, Head-Direction, and Spatial-View Cells. *PLOS Comput. Biol.* **3**, e166 (2007).

Supplementary statistics table

All tests involving multiple comparisons are Bonferroni corrected. All statistical tests are two-sided unless otherwise specified

Figure	Sample size (numbers indicate sessions unless otherwise stated)	Statistical test	Values
**Fig. 2b** Confidence interval for proportion of head movements during body movement or stops	Marmoset= <br>28200 head movements during body movement <br>113056 head movements during stops <br>141256 total head movements (n) <br>Rat= <br>11736 head movements during body movement <br>1874 head movements during stops <br>13610 total head movements (n)	Confidence interval for a population proportion	Marmoset proportion: 0.199 head movements during body movements, 99% (z= 2.576) confidence interval [0.1969, 0.2024] <br>0.8004 head movements during body stop, 99% (z= 2.576) confidence interval [0.7976, 0.8031] <br>Rat proportion: 0.8623 head movements during body movements, 99% (z= 2.576) confidence interval [0.8534, 0.8712] <br>0.1377 head movements during body stop, 99% (z= 2.576) confidence interval [0.1288, 0.1466] <br>$p \pm Z_{\alpha/2} \cdot \sqrt{\frac{p(1-p)}{n}}$
**Fig. 2c** Head movement peak velocity comparison between the marmoset and the rat,	Marmoset= 179904 head movements <br>Rat= 80871 head movements	Two sided Wilcoxon signed-rank test	**Marmoset**: median= 371.8 deg/s **Rat:** median= 264.6 deg/s <br>$p < 5 \times 10^{-324}$ z = 113.8

**Fig. 4a** Comparison between SIC of place and view for Subject C	n= 84 neurons	Two-sided Wilcoxon signed-rank test	SIC is z-scored<br><br>Median SIC view= 7.968 Median SIC place= 2.424<br><br>p= 1.834 x 10^{-10}<br>Z= 6.375
Comparison between SIC of place and shuffle distribution for Subject C	n= 84 neurons	Two-sided Wilcoxon signed-rank test	SIC is z-scored<br><br>Median SIC place= 2.4242 Median SIC shuffle= -0.077<br><br>p= 1.295 x 10^{-30}<br>Z= 11.502
Comparison between SIC of view and shuffle distribution for Subject C	n= 84 neurons	Two-sided Wilcoxon signed-rank test	SIC is z-scored<br><br>Median SIC view= 7.968 Median SIC shuffle= -0.097<br><br>p= 7.386 x 10^{-48}<br>Z= 14.534
Comparison between SIC of place and view for Subject P	n= 120 neurons	Two-sided Wilcoxon signed-rank test	SIC is z-scored<br><br>Median SIC view= 9.35 Median SIC place= 2.36<br><br>p= 3.123 x 10^{-24}<br>Z= 10.156
Comparison between SIC of place and shuffle distribution for Subject P	n= 120 neurons	Two-sided Wilcoxon signed-rank test	SIC is z-scored<br><br>Median SIC place= 2.36 Median SIC shuffle= -0.089<br><br>p= 2.051 x 10^{-48}<br>Z= 14.621
Comparison between SIC of view and shuffle distribution	n= 120 neurons	Two-sided Wilcoxon signed-rank test	SIC is z-scored<br><br>Median SIC view= 9.35 Median SIC shuffle= -0.08<br><br>p= 4.51 x 10^{-76}

for Subject P			Z= 18.458
**Fig 5e** Speed score comparison between putative interneurons and putative pyramidal cells	n= 127 interneurons, 204 pyramidal neurons.	Two-sided Wilcoxon signed-rank test	Translation speed (TS)<br>Median speed score interneuron=0.19<br>Median speed score pyramidal=0.04<br>$p = 3 \times 10^{-22}$<br>Z = 9.7<br><br>Angular head velocity (AHV)<br>Median speed score interneuron=0.22<br>Median speed score pyramidal=0.03<br>$p = 1.3 \times 10^{-25}$<br>Z= 10.5
