# Supplementary material for: Primacy of vision shapes behavioral strategies and neural substrates of spatial navigation in marmoset hippocampus
Source: Nat Commun. 2024 May 14;15:4053. doi: 10.1038/s41467-024-48374-2 (PMC11093997; doi:10.1038/s41467-024-48374-2)
Supplement: Supplementary file 4 — Reporting Summary [file 41467_2024_48374_MOESM4_ESM.pdf]

nature portfolio

Corresponding author(s): Julio Martinez-Trujillo

Last updated by author(s): Mar 14, 2024

Reporting Summary

Nature Portfolio wishes to improve the reproducibility of the work that we publish. This form provides structure for consistency and transparency in reporting. For further information on Nature Portfolio policies, see our Editorial Policies and the Editorial Policy Checklist.

Statistics

For all statistical analyses, confirm that the following items are present in the figure legend, table legend, main text, or Methods section.

n/a	Confirmed	
☐	☒	The exact sample size (*n*) for each experimental group/condition, given as a discrete number and unit of measurement
☐	☒	A statement on whether measurements were taken from distinct samples or whether the same sample was measured repeatedly
☐	☒	The statistical test(s) used AND whether they are one- or two-sided<br>*Only common tests should be described solely by name; describe more complex techniques in the Methods section.*
☐	☒	A description of all covariates tested
☐	☒	A description of any assumptions or corrections, such as tests of normality and adjustment for multiple comparisons
☐	☒	A full description of the statistical parameters including central tendency (e.g. means) or other basic estimates (e.g. regression coefficient) AND variation (e.g. standard deviation) or associated estimates of uncertainty (e.g. confidence intervals)
☐	☒	For null hypothesis testing, the test statistic (e.g. F, t, r) with confidence intervals, effect sizes, degrees of freedom and P value noted<br>*Give P values as exact values whenever suitable.*
☒	☐	For Bayesian analysis, information on the choice of priors and Markov chain Monte Carlo settings
☒	☐	For hierarchical and complex designs, identification of the appropriate level for tests and full reporting of outcomes
☐	☒	Estimates of effect sizes (e.g. Cohen's *d*, Pearson's *r*), indicating how they were calculated

Our web collection on statistics for biologists contains articles on many of the points above.

Software and code

Policy information about availability of computer code

Data collection	Neural data acquisition was accomplished using wireless telemetry (CerePlex Exilis,Blackrock Microsystems, Salt Lake City, UT); Motion capture tracking was done using a total of 14, cameras (Optitrack, Flex 13, Corvallis, OR).
Data analysis	Offline spike sorting was performed using Plexon (Offline Sorter, Plexon Inc., Texas, USA). All analyses were done using custom-built MATLAB scripts (Mathworks Inc, version 2021b) unless otherwise specified. Scripts used in this manuscript can be found here: https://github.com/DiegoPiza/3Dmarms. A MATLAB wrapper was used to calculate the generalized additive models using PyGAM (version 0.8.0) , a Python open-source resource. Matlab's FieldTrip (https://www.fieldtriptoolbox.org/ , version 20220104) toolbox was used to analyze local field potentials. Colormap BrewerMap was used to visualize LFP power spectrum. VR_EyeSignalClassification (https://github.com/JMTNeuroLab/VR_EyeSignalClassification) was used for analysis of eye movements.

For manuscripts utilizing custom algorithms or software that are central to the research but not yet described in published literature, software must be made available to editors and reviewers. We strongly encourage code deposition in a community repository (e.g. GitHub). See the Nature Portfolio guidelines for submitting code & software for further information.

Data

Policy information about availability of data

All manuscripts must include a data availability statement. This statement should provide the following information, where applicable:

- Accession codes, unique identifiers, or web links for publicly available datasets
- A description of any restrictions on data availability
- For clinical datasets or third party data, please ensure that the statement adheres to our policy

Source data are provided with this paper, and can be downloaded at https://figshare.com/s/b7b4a773c9a013880833 ; The complete dataset used in this study is available upon request to DBP or JCMT.

Research involving human participants, their data, or biological material

Policy information about studies with human participants or human data. See also policy information about sex, gender (identity/presentation), and sexual orientation and race, ethnicity and racism.

Reporting on sex and gender	N/A
Reporting on race, ethnicity, or other socially relevant groupings	N/A
Population characteristics	N/A
Recruitment	N/A
Ethics oversight	N/A

Note that full information on the approval of the study protocol must also be provided in the manuscript.

Field-specific reporting

Please select the one below that is the best fit for your research. If you are not sure, read the appropriate sections before making your selection.

☒ Life sciences ☐ Behavioural & social sciences ☐ Ecological, evolutionary & environmental sciences

For a reference copy of the document with all sections, see nature.com/documents/nr-reporting-summary-flat.pdf

Life sciences study design

All studies must disclose on these points even when the disclosure is negative.

Sample size	The location of the hippocampus in non-human primates (deep in the medial temporal lobe) increases the difficulty to successfully target this region with deep electrodes, success rate of surgeries was ~40%, which limited the number of subjects we could include in this work. We believe however, that the uniqueness of this dataset given the model organism, coupled with the high neuronal sample size(n= 331 single neurons) and the clear and reproducible effects across subjects, provides a robust foundation for our conclusions. This sample size is consistent with previously published work in non-human primate hippocampal physiology (2 subjects, 183 neurons) (Gulli et al. 2020)
Data exclusions	We excluded high amplitude artifacts (abrupt jumps in the signal that are of non-biological origin) as data outliers present during spike sorting and tracking data preprocessing (translation speed > 300cm/s, angular head velocity > 2000 deg/s). Spatial bins with less than 3 visits per session were excluded from the analyses since we considered these locations under-sampled for appropriate statistical analysis.
Replication	The main findings in this manuscript were independently found in both animal subjects.
Randomization	Randomization is not relevant to our study since there is only one experimental group.
Blinding	Blinding is not relevant to our study. The analyses and data collection methods conducted here have been reported in previous literature (see methods), and they have been designed to ensure objective collection and interpretation of all the data signals.

Reporting for specific materials, systems and methods

We require information from authors about some types of materials, experimental systems and methods used in many studies. Here, indicate whether each material, system or method listed is relevant to your study. If you are not sure if a list item applies to your research, read the appropriate section before selecting a response.

Materials & experimental systems

n/a	Involved in the study
☒	☐ Antibodies
☒	☐ Eukaryotic cell lines
☒	☐ Palaeontology and archaeology
☐	☒ Animals and other organisms
☒	☐ Clinical data
☒	☐ Dual use research of concern
☒	☐ Plants

Methods

n/a	Involved in the study
☒	☐ ChIP-seq
☒	☐ Flow cytometry
☒	☐ MRI-based neuroimaging

Animals and other research organisms

Policy information about studies involving animals; ARRIVE guidelines recommended for reporting animal research, and Sex and Gender in Research

Laboratory animals	2 adult (1 female, aged 5y and 1 male, aged 3y) common marmosets (Callithrix jacchus)
Wild animals	none
Reporting on sex	Both male and female subjects were included in this study, no sex-based differences were found.
Field-collected samples	N/A
Ethics oversight	The animal care and handling procedures, encompassing basic care, housing and husbandry, animal training, surgical procedures, and experiments (data collection), were granted approval by the University of Western Ontario Animal Care Committee. This approval guarantees adherence to federal (Canadian Council on Animal Care), provincial (Ontario Animals in Research Act), regulatory (e.g., CIHR/NSERC), and other national CALAM standards, ensuring the ethical use of animals. The animals' physical and psychological well-being was regularly assessed by researchers, registered veterinary technicians, and veterinarians.

Note that full information on the approval of the study protocol must also be provided in the manuscript.
